# Retention and wear assessment of novel PolyEtherKetoneKetone versus traditional cobalt chromium material used in digital double crown retained removable partial dentures

**DOI:** 10.1186/s12903-026-09411-2

**Published:** 2026-08-08

**Authors:** Rania Abd elsattar Mangood Ramadan, Sawsan Maged Fadl, Shaimaa Mohamed Abu El Sadat Ali, Rami Maher Ghali

**Affiliations:** 1 Oral and Maxillofacial Prosthodontics Department, Faculty of Dentistry, Ain shams university, Cairo, Egypt; 2 Oral and Maxillofacial Radiology Department, Faculty of Dentistry, Ain shams university, Cairo, Egypt

**Keywords:** Retention, Wear, PolyEtherKetoneKetone, Digital, Double crown, Partial dentures

## Abstract

**Background:**

Conventional removable partial dentures (RPDs) involve a process that is often susceptible to errors and demands a significant investment of time. The introduction of computer-aided design and computer-aided manufacturing (CAD/CAM) technology and materials witnessed significant advancements in recent years to produce accurate removable partial dentures. This study evaluated the retention and wear of PolyEtherKetoneKetone (PEKK) compared to Cobalt-Chromium (CoCr) in digitally fabricated double crown removable partial dentures for lower Kennedy Class I cases.

**Methods:**

The study was performed on 3D digital models of mandibular Kennedy Class I with prepared premolars bilaterally. The double crown RPDs were designed and fabricated digitally to get 14 copies of RPDs. Two groups were defined (*n* = 7); the first group were milled from Co-Cr discs, and the other’s from PEKK blanks. Initial retention was measured using a universal testing machine. RPDs were then subjected to dynamic loading in a chewing simulator over 100,000 cycles followed by 540 manual insertion and removal cycles and were remounted on the testing machine to measure final retention simulating six months. Wear was assessed using Geomagic Control X software, where negative deviations highlighted areas of degradation. Data was collected and analyzed for statistical analysis. Two Sample t-test was used to compare between the two groups and Paired t test to study the changes within each group. The significance level was set at *P* ≤ 0.05.

**Results:**

PEKK group showed a statistically significantly higher final retentive values after 6 months (11.6 N) than Co-Cr (2 N). Moreover, results showed higher retention loss in Co-Cr group (16.9 N) compared to PEKK (3.7 N). Additionally, wear assessment of primary copings indicated a higher deviation in the Co-Cr group (-0.25 mm) compared to PEKK (-0.096 mm), with a notable correlation (0.651, P-value = 0.011) between retention loss and wear.

**Conclusions:**

Within the limitation of this study, it could be concluded that PEKK provided digitally fabricated double crown RPDs with higher retention values and less wear than Co-Cr after simulation of 6 months of RPD use.

## Background

Traditional treatment of partially edentulous patients with mandibular distal extension removable partial denture has been associated with several problems related to aesthetics, retention, stability, masticatory efficiency, and so less patient satisfaction [1]. An alternative treatment, tooth-retained telescopic RPDs, can offer acceptable aesthetics and improved retention and stabilization due to the splinting action of abutments, which may be particularly beneficial for high-risk patients not suitable for implants [2].

The double crown system, also known as telescopic crown system, is composed of two copings; a primary inner coping cemented onto the abutment and a secondary outer coping attached to the denture [3, 4]. The prosthesis’s performance and longevity are greatly influenced by the material selection for the framework [5, 6]. In past, cobalt-chromium (CoCr) was the traditionally used material because of its considerable mechanical strength, high elastic modulus, and precise fit [7, 8]. However, it has some disadvantages like poor aesthetics with metal display, adverse tissue reactions, and biofilm production [7, 8]. Recently, the dental industry has focused on Poly-aryl-ether-ketone (PAEK) polymers, particularly Poly-ether-ketone-ketone (PEKK), that is located at the peak of the thermoplastic’s quality pyramid. PEKK is known for its superior mechanical properties with 80% higher compressive strength compared to Poly-ether-ether-ketone (PEEK) [9, 10]. It also demonstrates excellent shock absorption (approximately 65 MPa) and high fracture resistance properties, that enhance its ability to withstand functional loading. Notably, its compressive strength (≈ 246 MPa) is close to that of dentin (≈ 297 MPa), supporting its suitability for intraoral load-bearing conditions. Furthermore, the elastic modulus of PEKK is comparable to cortical bone, promoting more favorable stress distribution and as a result, it can be used as a prosthetic biomaterial [11–13]. PEKK also offers a stable dimensional structure at high temperatures, excellent mechanical and chemical resistance to wear, making it an attractive option for RPDs frameworks and can produce promising functional and esthetic results [14, 15]. Moreover, Clinically, favorable outcomes have been reported; as PEKKton framework has been successfully utilized both in implant-supported prostheses for the fully edentulous maxilla and in tooth-supported fixed prostheses in partially edentulous mandibular cases, demonstrating promising functional and esthetic results [16]. However further studies are needed as there are no long-term observation and enough clinical data found.

Traditionally, RPDs involved several complicated steps starting from manual impressions to framework fabrication using lost wax technique. Recently, CAD/CAM technology has been widely used for fabrication of various dental prostheses to enhance the repeatability of prostheses production and reduce inter-operator variation [17, 18]. Furthermore, frameworks are properly refined exhibiting minimal porosity which contributes to the enhanced strength, precision, and fit of the resultant prostheses [19–21].

Ensuring proper retention and high wear resistance of RPD frameworks is critical for clinical success and reducing clinical complications [22–26]. The aim of this study was to assess the retention and wear properties of CAD/CAM digital CoCr and PEKK double crown RPD frameworks to provide useful information about their performance in Kennedy class I cases. The assessment utilized advanced techniques and measurement methods to ensure accurate and reliable results. The null hypothesis predicted that there would be no significant differences between CoCr and PEKK digitally fabricated double crown RPD frameworks regarding the retention and wear.

## Methods

This study was performed on mandibular Kennedy Class I models and the abutments were first and second premolars bilaterally. Two types of materials were used to fabricate CAD/CAM milled double crown retained RPDs. Thus, two equal groups were defined; Co-Cr group: the copings and RPD framework were milled from Co-Cr, while PEKK Group: they were milled from PEKK. Sample size calculation was performed using a power analysis software program (G*Power version 3.1.9.7; Heinrich Heine University Dusseldorf). The analysis was designed to have adequate power to apply a two-sided statistical test of the null hypothesis that there is no difference between tested groups. By adopting an alpha level of (0.05) a beta of (0.2) i.e. power = 80% and an effect size (d) of (1.63) calculated based on the results of a previous study [27]; the predicted sample size (n) was a total of (14) samples.

### Construction of 3D printed casts and mucosa simulation

A master mandibular stone model of Kennedy class I was scanned utilizing a 3shape desktop scanner (E2; 3Shape A/S), which offers a scanning accuracy of ≤ 10 μm ensuring high precision for digital modeling. A multi-view rotational scanning protocol was applied to capture all surfaces and then virtualized into a digital model. The abutment teeth were digitally prepared using Exocad software (Exocad DentalCAD 2.4 Plovdiv, Exocad GMBH). A uniform axial reduction of 2–3 mm was applied to each abutment tooth using the virtual preparation tool, followed by a uniform occlusal taper set using the digital surveying module to define a common path of insertion. The finish line was configured as a chamfer, with a uniform 1.5 mm width, using the software’s margin designer tool [15]. These parameters were applied identically to ensure preparation standardization and eliminate operator-dependent variability. To simulate mucosal conditions, the edentulous areas were selected on both sides then “reduction tool” was used in the designing software to vertically cut back 2 mm thickness at the ridges to create a space for mucosa simulation. The designed model was then imported into the software (Chitubox V1.9.1, Chitubox) of 3D printing device (Anycubic Photon Mono X resin SLA 3D printer, Anycubic Technology CO.) to be arranged by using the “slice” tool and adding support arms. Then, fourteen identical 3D hollow casts were printed from curable resin (HARZ Labs model resin, HARZ Labs LLC). Finally, a vacuum sheet was pressed over the designed space for mucosa simulation, and mucosa gingival mask was injected and pressed.

### Construction of primary copings

**Fig. 1:**
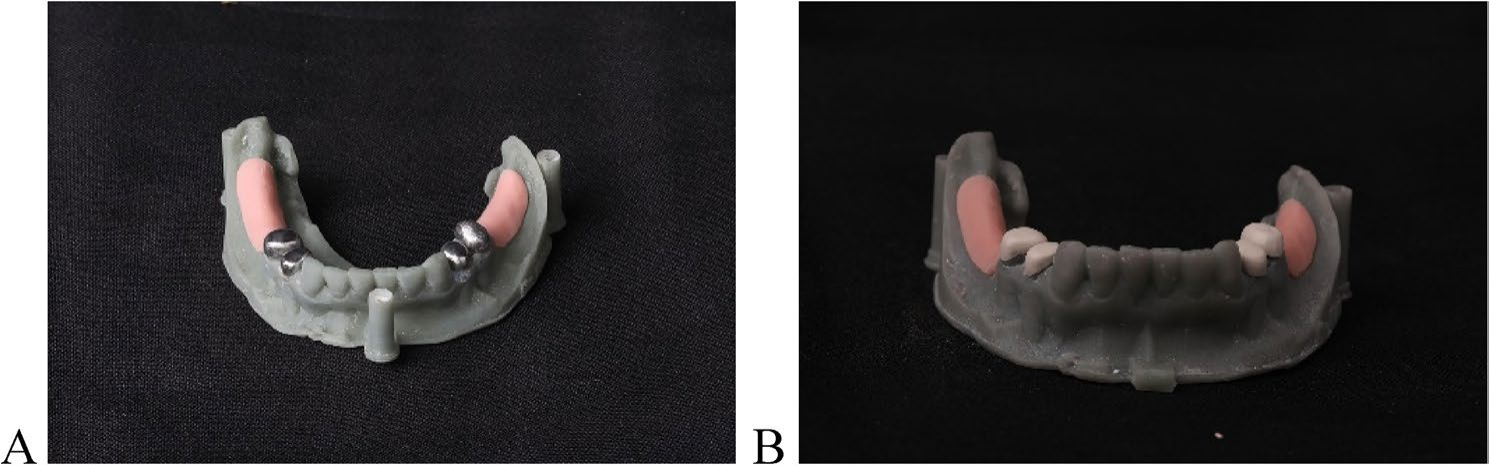
Trial fitting of primary copings on the model. **A**, Co-Cr primary copings. **B**, PEKKton primary copings

The double crown RPDs were designed using Exocad software (Exocad DentalCAD 2.4 Plovdiv, Exocad GMBH). Four abutments were chosen to begin the design for each model, and a telescopic design order was chosen. Digital surveyor tool was employed to establish a uniform path of insertion and same parameters for designing primary copings were employed for all models to include a 1 mm vertical parallel band height from the finish line, a 4-degree occlusal taper, and a minimum material thickness of 0.6 mm. The STL file was then sent to 5-axis milling machine (inLab MC X5, Dentsply Sirona), and support arms were added utilizing the milling system’s nesting software which allowed consistent orientation, angulation, and thickness preservation across all samples. For each model, 4 sets of primary copings were milled; Co-Cr group were wet-milled from 20 mm Cobalt chromium disc (remanium star *MD II*,* Dentaurum*) using carbide metal-specific carbide burs, while PEKK group were dry-milled from 20 mm PEKKton blank (Pekkton Ivory – Cendres+MMétaux Medtech) using polymer-specific milling burs. Dedicated tool sets and milling strategies were employed for each material to ensure precision, avoid cross-contamination, and comply with manufacturer recommendations. For standardization, the primary copings were minimally finished and polished at the support arms areas. The copings were then seated on their casts, sandblasted, and cemented to the abutment teeth using self-adhesive resin cement (Theracem automix, Bisco) (Fig. 1).

### Construction of secondary copings and RPD frameworks

**Fig. 2:**
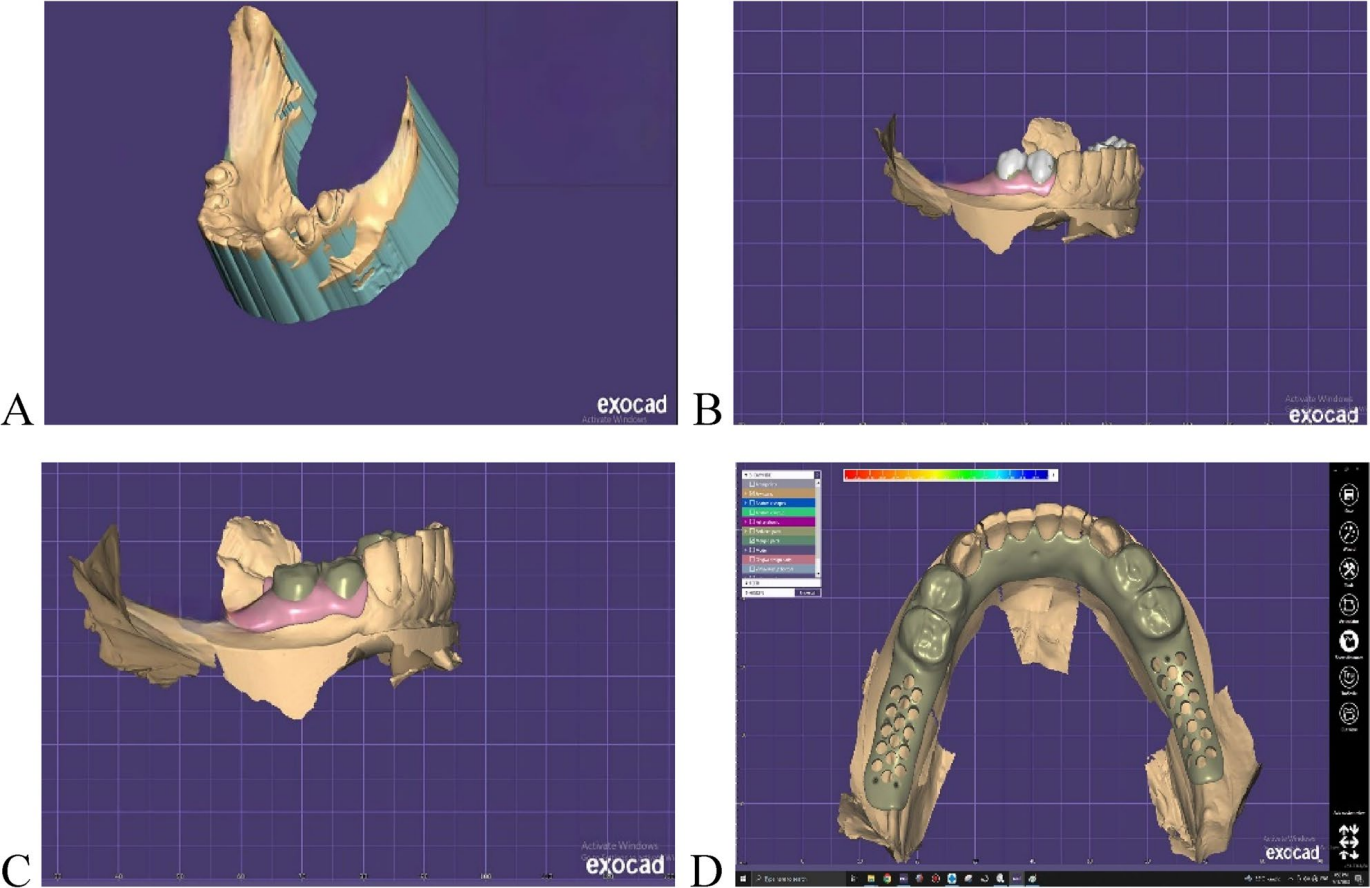
RPD double crown framework design. **A**, Digital surveying and blocking out the undercuts. **B**, Secondary copings with anatomical teeth forms. **C**, Secondary copings with cut back of 1.2 mm. **D**, Completed RPD design joined to secondary copings

**Fig. 3:**
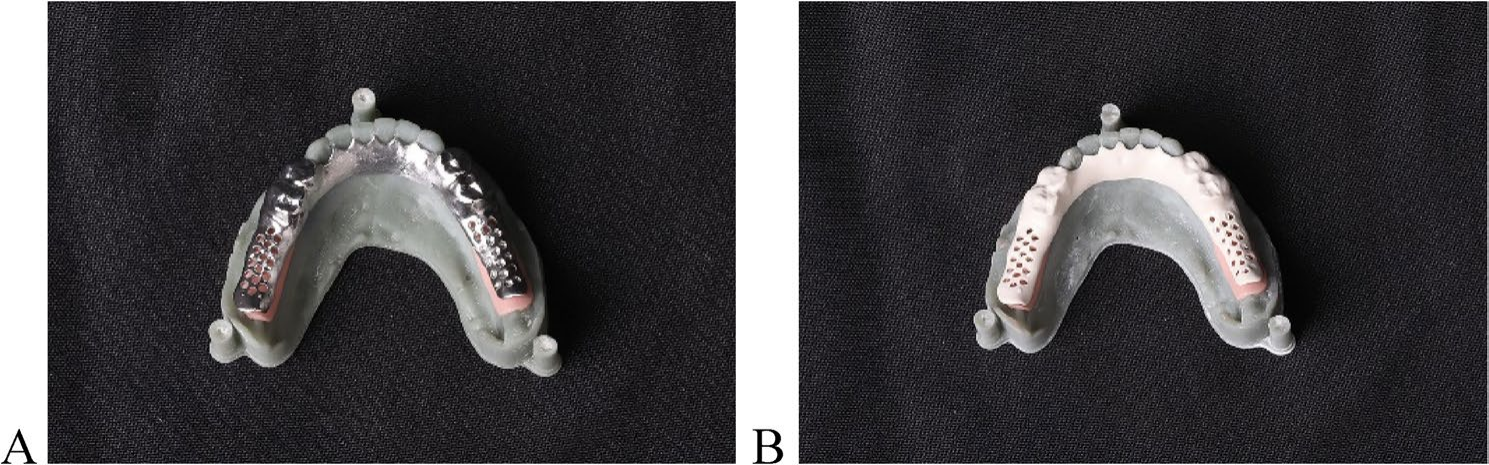
Seating of RPD framework on the primary copings on the model. **A**, Seating of Co-Cr RPD framework. **B**, Seating of PEKKton RPD framework

A partial denture design order was chosen to design the secondary copings, denture bases, and mandibular major connector. The path of insertion was identified using the digital surveyor and the software library was used to select anatomical teeth forms, which were adjusted and adapted to the finish lines, then a 1.2 mm cutback was performed to accommodate the veneering material. The RPD was then designed based on traditional principles by selecting the meshwork form and connecting the bases by lingual plate major connector and then joining the framework to the secondary copings with the sculpting tool (Fig. 2). The STL file of double crown framework design was exported and nested by the same milling machine’s nesting software. The same materials were used as previously mentioned in manufacturing primary coping (Fig. 3). Following that the acrylic teeth (Acrostone plus teeth A2 shade, Acrostone) were set and the RPDs were then processed with self-cured acrylic resin (Vertex regular acrylic denture base, Vertex-Dental) in the usual manner.

### Evaluation of retention and wear

For both groups, the outer surfaces of the primary copings and the inner surfaces of secondary copings were initially scanned using the 3Shape desktop scanner after applying a scannable spray (SHERAscan spray; SHERA Werkstoff-Technologie GmbH & Co) to the Co-Cr group, then STL files were obtained to represent the reference data for further wear evaluation.

**Fig. 4:**
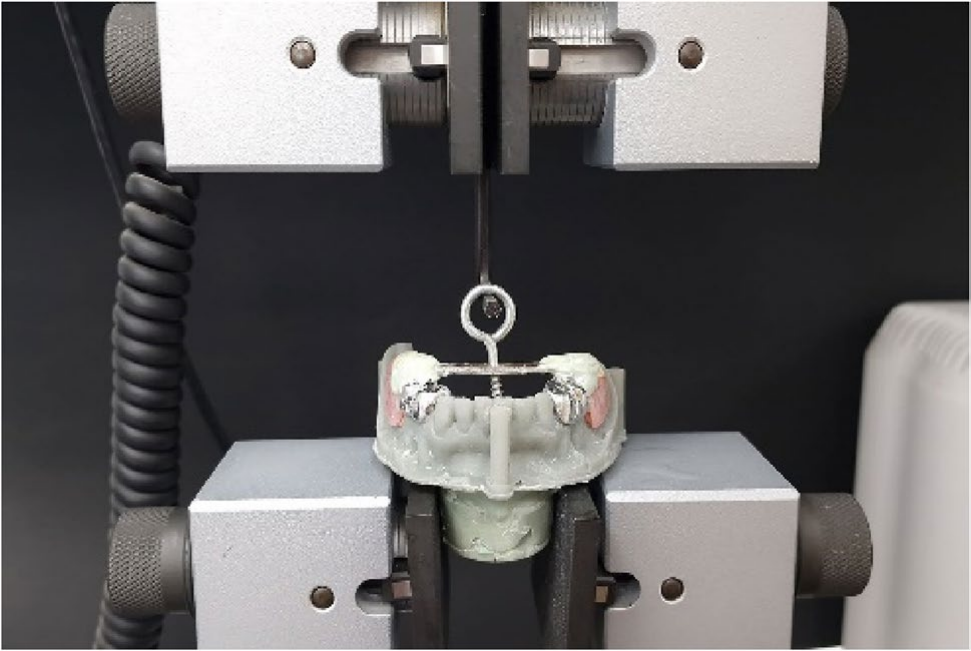
Occlusal plane is parallel to the base of the universal testing machine

Each RPD model was equipped with a cylindrical acrylic projection and a horizontal metal plate, which was secured with a metal hook for attachment to a universal testing machine (INSTRON 3365). The occlusal plane of the RPD was adjusted to be parallel to the base of the testing machine to ensure vertical alignment during dislodgment, and the model was attached to the lower compartment of the machine using the previously constructed cylindrical acrylic projection. This machine was used to exert a preload of 50 Newtons for 20 s, maintaining a uniform crosshead velocity of 50 mm/min to measure the initial retention force necessary to detach the RPD from the primary copings (Fig. 4) [21, 27].

**Fig. 5:**
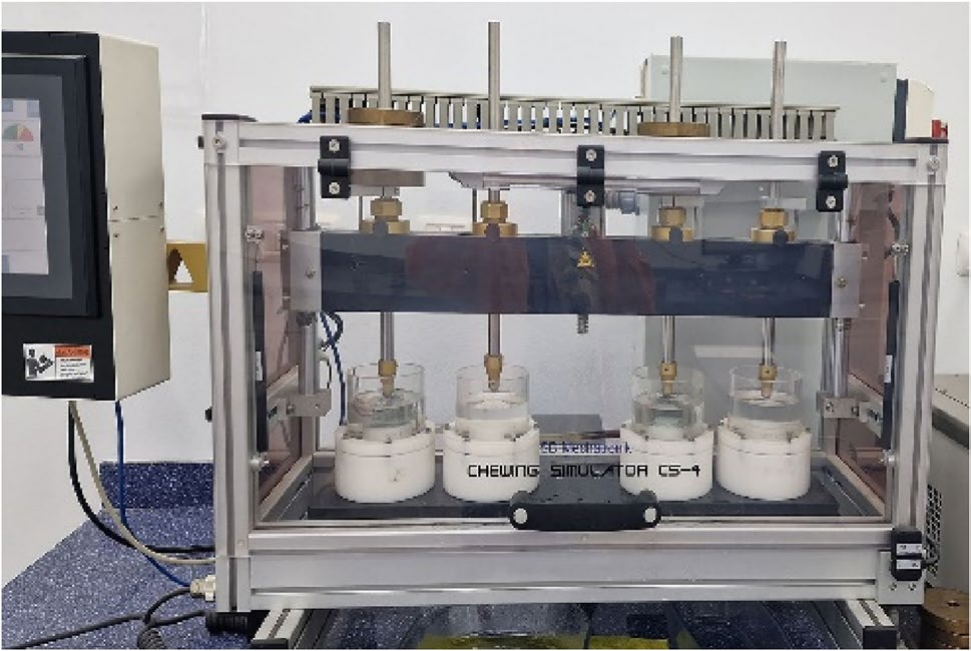
CS-4.4 - SD Mechatronic chewing simulator

Following initial testing, the models underwent a chewing simulation (CS-4 Robota Model ACH-09075DC-T) for a total of 100,000 cycles to mimic six months of functional use (Fig. 5) [28–30]. Each group underwent chewing simulation under identical conditions, including a specimen chamber filled with artificial saliva and load settings established at 50 N through the steel stylus of the machine that acts against a metal plate fixed to the RPD framework [31]. The software parameters were programmed to a speed of 60 mm/sec, a vertical movement of 2 mm, a lateral movement of 0.7 mm, and a frequency of 1.6 Hz. The antagonistic material was the steel stylus of the machine that acts against a metal plate fixed to the RPD framework. Each RPD was then manually inserted and removed 540 times manually by a trained operator to simulate daily use for hygienic purposes over the same period [4, 32, 33]. Each insertion was performed with firm finger pressure until full seating was achieved, and removal was completed in a vertical direction, simulating typical patient handling during hygiene practices. After that, the final retention force was measured again using the universal testing machine.

**Fig. 6:**
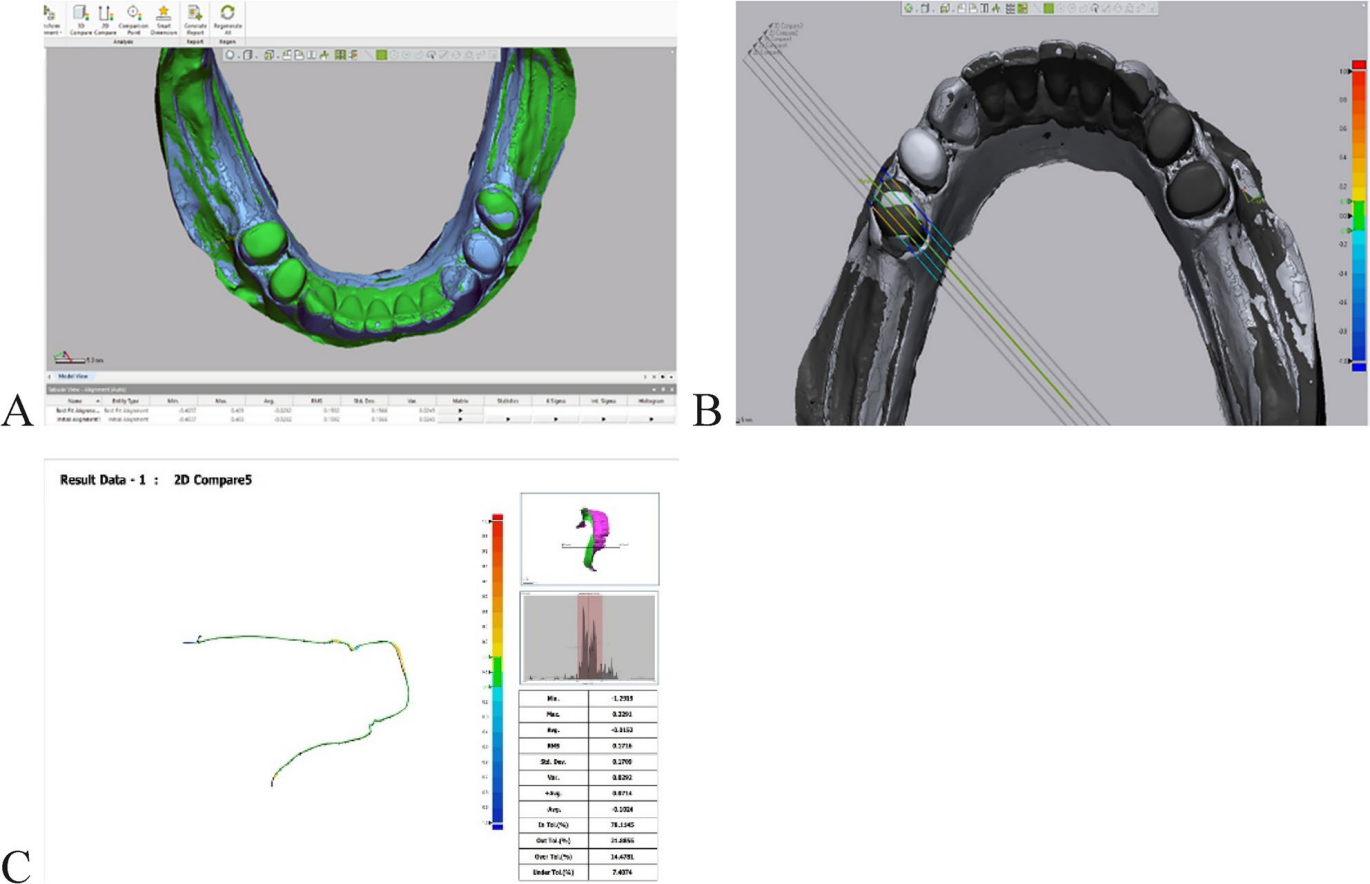
Wear assessment of the primary copings using Geomagic control X. **A**, Alignment of the reference and measured data of the primary copings. **B**, The consecutive sections through the 2nd left premolar area. **C**, 2D compare of one of the sectional cuts showing the deviation between the primary copings before and after function simulation

**Fig. 7:**
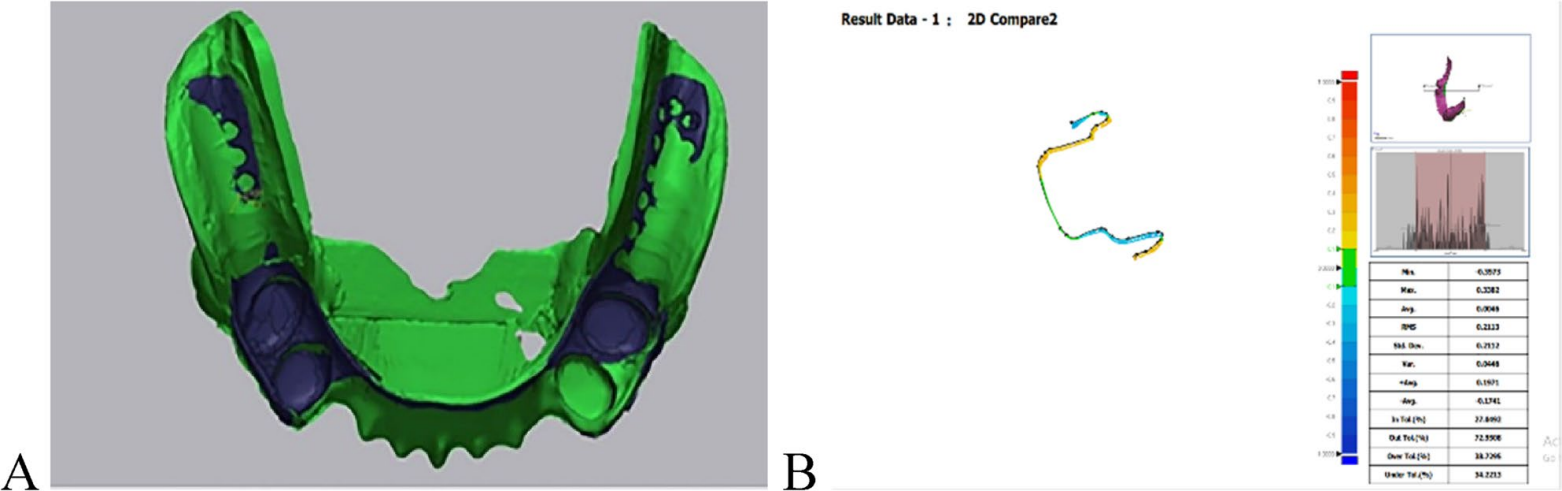
Wear assessment of the secondary copings using Geomagic control X. **A**, Alignment of the reference and measured data of the secondary copings. **B**, 2D compare of one of the sectional cuts showing the deviation between inner surface of secondary copings before and after function simulation

The surfaces of primary and secondary copings were scanned again after simulation using the same scanner to represent STL files of the measured data for wear evaluation. To evaluate the deviations of the copings before and after function simulation, a 3D assessment software (Geomagic Control X, Version 2023.2.0, 3D Systems Inc) was utilized. For each evaluation, two STL files were imported, one representing the reference data and the other representing the post-simulation measured data. Alignment of the data was done first by the initial alignment based on shared geometry and then by the best-fit alignment using iterative closest point (ICP) algorithm. Then, a deviation map was generated to visually represent the differences between the two data. To perform analysis, a reference plane was drawn to divide the coping equally into mesial and distal halves then five consecutive equidistant longitudinal sections with 1 mm interval were acquired for each coping area. The deviation map for each 2D compare was analyzed and areas of material loss or wear were identified with negative deviations (Figs. 6 and 7).

wear assessment of the primary copings using Geomagic control X. A, Alignment of the reference and measured data of the primary copings. B, The consecutive sections through the 2nd left premolar area. C, 2D compare of one of the sectional cuts showing the deviation between the primary copings before and after function simulation.

### Statistical analysis

Statistical analysis was performed using R programming language with the R studio IDE including the code in R script. Data were presented as median and range values. Two Sample t-test was used to compare between the two groups and Paired t test to study the changes within each group. Generalized linear model with Gaussian family was used to study the changes of the deviations after 6 months of the two groups as the group is combined with the status of copings. The significance level was set at *P* ≤ 0.05.

## Results

Numerical data were explored for normality by checking the distribution of data and using Shapiro-welk test of normality. The results of this test yielded both p-values exceeded the alpha threshold of 0.05, indicating that the data in both groups follows a normal distribution. Then, the homoscedasticity of data was checked. Although the data were normally distributed, median and range values were presented for descriptive purposes to provide a robust summary of the data that is less influenced by potential outliers or extreme values, which can sometimes occur even in normally distributed datasets.

Regarding the retention, statistical analysis revealed a significant difference in the initial and the final retentive forces (after 6 months) between the two groups. The Co-Cr group exhibited lower final retention compared to PEKK although the initial retention was higher in this group as detailed in Table 1. There was a statistically significant difference in initial retention values between the Co-Cr and PEKK groups (*t*(12) = 3.1, *p* = 0.0088), and a large effect size (Cohen’s *d* = 1.67, 95% CI [0.41, 2.88]). After six months of simulated function, the retention values remained significantly different between groups (*t*(12) = − 22.2, *p* < 0.001), and an extremely large effect size (Cohen’s *d* = − 11.85, 95% CI [− 16.64, − 7.05]).

**Table 1:** Median, range values and results of Standard Two Sample t-test of the two groups at the initial retention (N) and retention after 6 months (N)

	Co-Cr Group			PEKK Group			*P*-value
	Median	Min	Max	Median	Min	Max	
Initial Retention (N)	18.9	16.2	22.2	14.4	13.6	19.9	(*P* = 0.008)
Retention After 6 Months (N)	2.0	1.5	2.9	11.6	9.9	12.7	(*P* < 0.001)

There was also a statistically significant difference in retention loss over six months between the groups (*P* < 0.001) with the Co-Cr group experiencing greater retention loss than the PEKK, (*t*(12) = 17.3, *p* < 0.001), and a very large effect size (Cohen’s *d* = 9.25, 95% CI [5.44, 13.03]) as outlined in Table 2.

**Table 2:** Median, range values and results of Paired Two Sample t-test for retention loss after 6 months (N) of the two groups

	Median	Min	Max	*P*-value
Retention Loss(N) Co-Cr Group	16.9	19.3	14.7	(*P* < 0.001)
Retention Loss(N) PEKK Group	3.7	6.9	2.9	

Regarding the wear, statistical analysis showed a statistically significant difference of negative value deviations after 6 months of the primary copings between the two groups (*P* = 0.020). The primary copings of Co-Cr group demonstrated more wear compared to those in the PEKK, as presented in Table 3. Conversely, for the secondary copings, there was no statistically significant difference of negative value deviations after 6 months between the two groups (*P* = 0.780).

**Table 3:** Median, range values and results of generalized linear model for the negative values deviation after 6 months (mm) of the two groups in both the primary and secondary copings of each group

Negative value deviation (wear) after 6 months (mm) 1ry copings	Median	Min	Max	*P*-value
Co-Cr Group	-0.25162	-0.1198	-0.55836	0.020
PEKK Group	-0.09618	-0.0369	-0.17072	

**Negative value deviation (wear) after 6 months (mm) 2ry copings**	**Median**	**Min**	**Max**	***P*** **-value**
Co-Cr Group	-0.1589	-0.0981	-0.9201	0.780
PEKK Group	-0.22882	-0.1906	-0.48862	

**Fig. 8:**
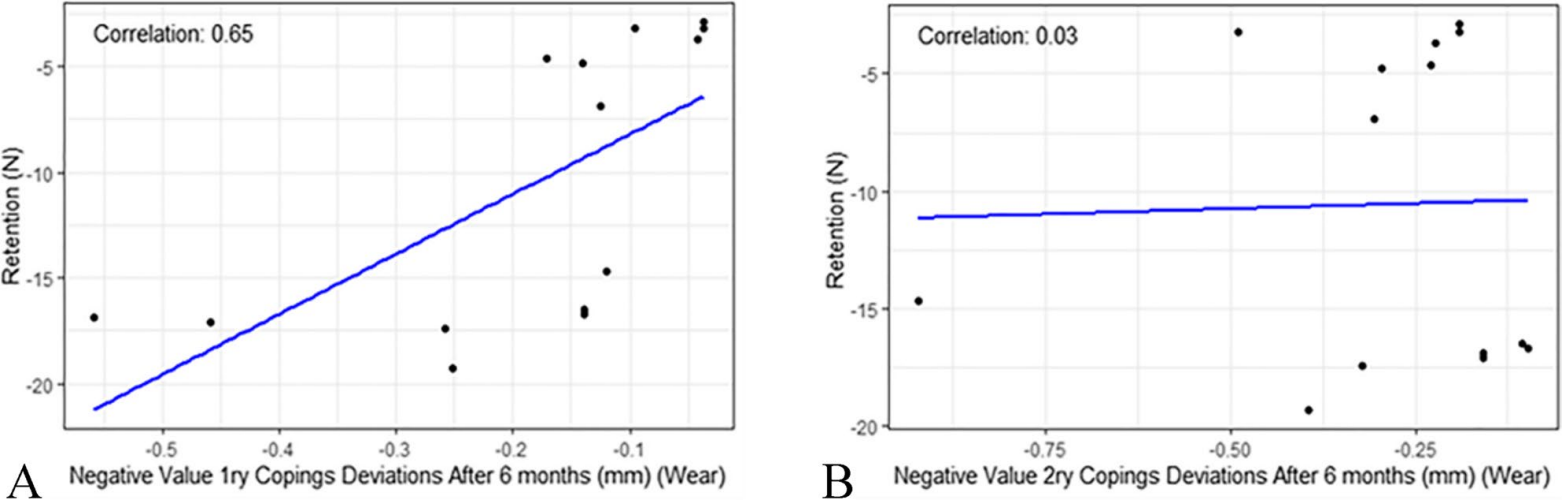
Scatter plot with linear model shows correlation between negative value deviation of copings and retention loss after six months in the two groups: (**A**) primary copings, (**B**) secondary copings

Correlation analyses were performed using Pearson’s correlation coefficient to explore associations between negative wear values and retention differences. Analysis was conducted for the entire cohort and stratified by group and clinical status (primary copings vs. secondary copings). A statistically significant correlation between the primary copings wear and retention loss after 6 months was found (Correlation coefficient = 0.651, *P*-value = 0.011) (Fig. 8a). The significance was in PEKK group. While regarding the secondary copings, there was no statistically significant correlation within the two groups (Correlation coefficient = 0.028, *P*-value = 0.921) (Fig. 8b).

## Discussion

This study was done to analyze two contemporary materials (Co-Cr and PEKK) used in digital manufacturing of telescoping crowns. These combinations of materials can result in different resistance to repetitive removal and insertion cycles and varying surface wear patterns.

In this study, retention was assessed using vertical dislodgement forces measured with a universal testing machine. This testing approach is commonly employed in in-vitro prosthodontic research as it provides a standardized, reproducible, and controlled method for comparing materials under identical loading conditions. Since the universal testing machine can only follow intermittent movements in one vertical plane and generates static loads only so the chewing simulator device was used to imitate the lateral forces acting on the abutments during function and to simulate the chewing cycles [31–33].

The number of chewing cycles in this study was 100,000 cycles which was based upon the average number of chewing cycles expected after 6 months of function. Moreover, the initial wear that is the most important feature of the cylindrical telescopic system usually occurs in the first 5,000 cycles. So the study was relevant to simulate the initial wear plus a safety margin up to the 100,000 cycles [28–30].

To simulate the oral environment, artificial saliva was utilized throughout the testing process, and the removable partial dentures (RPDs) were subjected to 540 cycles of repeated insertion and removal to replicate six months of clinical wear. This number of cycles was calculated based on the standard clinical assumption that a patient inserts and removes their prosthesis three times daily for routine hygienic maintenance. Using this established frequency, the 540-cycle protocol provides a standardized, accelerated simulation of the mechanical fatigue and material wear experienced by the RPD during regular, short-term daily use [33].

The results revealed significantly higher final retentive values after 6 months of function simulation in PEKK group than CoCr group, thus the null hypothesis was rejected. However, it was found that the Co-Cr group has a higher initial retention value than PEKK, which may be important in the initial phase of adaptation to a new prosthesis. The observed decrease in retention of CoCr group suggested that the material underwent changes over time and exhibited wear that lowered the precision fit of the double crown components over time, which could impact the final retention [5]. While the higher final retention observed with PEKK group might be associated with the material’s intrinsic properties [14, 24]. In particular, PEKK demonstrates notable resistance to wear and corrosion, which may help preserve its surface integrity and maintain a consistent fit over time. Moreover, the polymer’s mechanical properties and stability contribute to sustained retention and its long-term performance [14, 24].

It has been recommended that the retentive forces should be maintained at a level that does not harm the bone. Additionally, it is advised that retentive forces for double crown retained RPDs remain between 5 and 10 N. Although the retentive forces in PEKK group were higher, the final retentive force measurements fell within the recommended parameters [24].

The results showed that the CoCr group experienced a greater reduction in retention following a simulated 6 months of function compared to the PEKK group. This reduction in retention could be attributed to metal abrasion on the coping surfaces after functional use [5]. While the flexibility and modulus of elasticity of PEKK make it ductile material that yields and adapts well, thereby ensuring a superior marginal fit and mechanical compatibility between the two copings following functional use [4, 14, 24, 25].

It has been suggested that the external surfaces of the primary copings and the internal surfaces of the secondary copings of the double crown retained RPD are susceptible to elastic reversible deformations and plastic irreversible deformations during the processes of insertion, removal, and masticatory actions [23]. Upon comparing the deviation of the double crown retained RPD after 6 months use, negative values were considered as they indicated the wear or material loss that occurred in this period [22]. Geomagic Control X software was selected to evaluate the scanned data as it has a 2D compare tool that is used to provide valuable insights into the wear patterns and changes in dental prosthetic components. Moreover, it is known for its accuracy in measuring and analyzing deviations of 3D surfaces and objects and thus provides reliable and precise data [17, 19].

Regarding wear of primary copings, the CoCr group showed statistically significantly higher values than PEKK group. The deviation maps revealed that Co-Cr copings showed localized wear, especially at inner axial walls, likely from frictional abrasion during repeated insertion. While, PEKK copings demonstrated more diffuse and uniform wear, possibly due to its higher elasticity and energy-absorbing behavior, which reduces point loading and suggests uniform stress distribution. This difference in wear patterns may be due to PEKK’s lower elastic modulus, higher shock absorption (65 MPa), and reduced hardness that allows it to distribute stresses more evenly, minimizing concentrated wear.

The differences in wear between PEKK and CoCr may be attributed to the unique mechanical properties of each material, including hardness, surface finish, and resistance to friction [22]. The higher wear exhibited by CoCr primary copings implies that they may be prone to abrasion or surface degradation when subjected to mechanical stress during mastication. It was found that when metal alloys come into contact and experience friction against other metals, the surface of the metal alloys undergoes significant adhesive wear [4]. Conversely, the lower wear values observed with PEKK primary copings suggests that PEKK, as a polymer material, may be more resistant to wear and abrasion compared to Co-Cr [14, 23]. Moreover, it could be explained by the elasticity and adaptability of polymer materials as postulated by a Kotthaus et al. [15] which reported that PEKK–PEKK combination remained constant during aging.

Regarding wear of secondary copings, the results obtained revealed no statistically significant difference between the two groups. It was found that secondary copings constructed with a 4-degree occlusal taper can provide a slight resiliency that facilitates prosthesis insertion and helps to prevent the material excessive wear [24, 26]. Moreover, it was postulated that the wear of the secondary and especially primary copings possess a major challenge for clinicians. Although secondary copings can be replaced with relative ease, the replacement of primary copings necessitates the removal of the crowns, potentially leading to damage to the residual tooth structure [23].

The results of this study showed a statistically significant correlation between loss of retention and wear regarding the primary copings of CAD/CAM double crown RPD. This finding suggests that as primary copings experience wear or surface loss over time, there is a noticeable reduction of their retention properties [5, 20]. While regarding the secondary copings, the results indicated that there is no statistically significant correlation between loss of retention and wear. It was found that secondary copings may exhibit a certain level of resilience or adaptability when it comes to wear, without significantly compromising their retention abilities [6, 21, 23].

The superior retention and reduced wear observed in the PEKK group can be attributed to its unique mechanical profile, specifically its low modulus of elasticity (approx. 5.1 GPa). Unlike the rigid Cobalt-Chromium (Co-Cr) alloy, PEKK’s high resilience allows for a ‘dampening effect’ under functional loads, which minimizes stress concentrations at the interface [34]. This elasticity, coupled with a low coefficient of friction, reduces the abrasive wear on the primary copings, whereas the high hardness of Co-Cr leads to faster degradation of the contact surfaces over time [35].

The clinical significance of this study reveals that PEKK dentures not only enhance durability and functional stability over time compared to CoCr dentures but also could improve patient comfort and satisfaction due to reduced denture movement. This stability potentially would reduce the need for ongoing maintenance and associated costs. While the current in vitro simulation demonstrates superior retention and reduced the wear of primary coping in PEKK compared to Co-Cr, these findings suggest favorable long-term clinical trajectories. Specifically, the maintenance of retentive values indicates that PEKK-based telescoping crowns may offer patients greater prosthetic stability over extended periods, reducing the frequency of clinical maintenance. Furthermore, the significantly lower wear observed on primary copings suggests that PEKK may better preserve the integrity of the abutment teeth compared to the more abrasive Co-Cr. Future longitudinal clinical trials are necessary to confirm if these mechanical advantages translate to improved patient-reported quality of life and reduced biological complications over years of functional loading.

While the current 6-month simulation provides a robust mechanical baseline, the long-term clinical performance of PEKK in the oral environment may be influenced by additional factors. High-performance polymers like PEKK are subject to hydrothermal aging; continuous exposure to saliva and fluctuating oral temperatures (thermocycling) can lead to slight water absorption, potentially affecting the material’s dimensional stability and retentive behavior over several years. Furthermore, while PEKK demonstrated superior wear resistance against the primary copings in this study, its performance against opposing natural dentition or abrasive food boluses must be considered. In a clinical Removable Partial Denture (RPD) scenario, the lower surface hardness of polymers compared to metallic alloys may lead to occlusal wear or ‘flattening’ of the secondary crown over extended periods of mastication. Chemical degradation, including exposure to acidic beverages or denture cleansing agents, may also alter surface roughness of PEKK, potentially increasing plaque stagnation or affecting the friction-fit interface. Therefore, while PEKK shows significant promise for digital manufacturing of telescoping crowns, longitudinal studies are required to assess its chemical and abrasive stability in the complex, dynamic oral environment.

These findings can guide clinical decisions to the long-term success of dental prosthesis by considering wear-resistant materials and design strategies for primary copings, whereas secondary copings may exhibit a degree of adaptability that allows for some wear without compromising retention. This supports broader clinical application of PEKK for long-term patient satisfaction and reduced prosthesis maintenance.

Some considerations to the clinical handling, repairability, and cost considerations of PEKK compared to Co-Cr. PEKK frameworks enable quick chairside correction with tungsten carbide burs and dependable cohesive chemical bonding using methyl methacrylate (MMA)-based primers when structural changes, tooth additions, or realignments are required due to hard or soft tissue remodeling. Co-Cr frameworks, on the other hand, rely only on mechanical retention and require intricate laboratory laser welding for repairs because they have no chemical affinity for acrylic resin. Practitioners must carefully consider the financial consequences despite the obvious clinical and patient-reported benefits of PEKK, such as decreased framework weight and enhanced gingival margin aesthetics. Because PEKK-based RPDs require specialized software parameters and premium CAD/CAM milling blanks, their initial production costs are still significantly higher than those of Co-Cr. Nonetheless, literature implies that this upfront financial investment is offset by a marked reduction in post-insertion complications, fewer unpaid clinic visits for sore spot relief, and lower lifetime maintenance overhead. Therefore, while Co-Cr remains a highly dependable option for patients with sound periodontal support prioritizing lower initial costs, PEKK telescopic attachments offer a superior, biomimetic alternative for the preservation of vulnerable natural dentition.

The limitation of this study was that it conducted for a short period of simulation of RPD use (6 months), so further studies are recommended to evaluate the correlation between changes in retentive force values and surface changes. Although the simulation of six months of use gives a valuable baseline for immediate clinical response; however, long-term clinical performance remains unproven. Expanding the scope to include multi-year expectations—accounting for material fatigue and chronic tissue adaptation—will significantly enhance clinical applicability. Also, retention measurement was relied solely on vertical dislodgement using a universal testing machine. The in-vitro nature of the experiment could fail to fully replicate the complex intraoral environment; specifically, the absence of thermal and humidity cycling that may overlook the potential for hydrothermal degradation. As the study utilized standardized models, it cannot account for the vast variability in clinical conditions, such as diverse ridge morphologies, irregular paths of insertion, and the lubricating or corrosive effects of human saliva. Although limited to small sample size *n* = 7 per group, a large effect size (1.63) derived from prior literature [27] enabled sufficient statistical power (80%). However, A larger sample size in future studies would be beneficial to further validate these mechanical trends and ensure that the observed differences in wear and retention are fully representative of a wider range of manufacturing variables.

Additional research and clinical trials are also needed to explore other combinations of materials that are used to retain PEKK double crown retained RPD.

## Conclusions

Within the limitations of this study, it could be concluded that in CAD/CAM double crown retained RPD, PEKK provides higher retentive force values and lower wear than Co-Cr after simulation of 6 months of RPD use, so PEKK/PEKK double crown RPD is considered suitable material combination to retain RPD.

## Data Availability

The data will be available from the corresponding author upon request.

## Abbreviations

RPDs: Removable partial dentures
PEKK: PolyEtherKetoneKetone
CoCr: Cobalt-Chromium
PAEK: Poly-aryl-ether-ketone
PEEK: Poly-ether-ether-ketone
CAD/CAM: Computer-aided design and computer-aided manufacturing
